# Associations of adolescent obesity with hypertension, diabetes mellitus and polycystic ovaries in Arabs and Jews in Israel—a nationwide study

**DOI:** 10.3389/fpubh.2024.1443756

**Published:** 2024-12-12

**Authors:** Yulia Treister-Goltzman, Dan Nemet, Idan Menashe

**Affiliations:** ^1^Department of Family Medicine and Siaal Research Center for Family Practice and Primary Care, The Haim Doron Division of Community Health, Faculty of Health Sciences, Ben-Gurion University of the Negev, Beer-Sheva, Israel; ^2^Clalit Health Services, Tel Aviv, Israel; ^3^Child Health and Sports Center, Meir Medical Center, Tel Aviv University School of Medicine, Tel Aviv, Israel; ^4^Department of Public Health, Faculty of Health Sciences, Ben-Gurion University of the Negev, Beer-Sheva, Israel

**Keywords:** adolescent obesity, severe obesity, hypertension, diabetes mellitus type 2, polycystic ovaries, comorbidities

## Abstract

**Background:**

Previous studies on the association of adolescent obesity with comorbid diseases in Israel were conducted predominantly in the Israeli Jewish population.

**Goal:**

To compare associations of adolescent obesity with Hypertension (HTN), Diabetes Mellitus type 2 (DM2), and Polycystic ovaries (PCO), singly or in combination, between Arabs and Jews in Israel.

**Methods:**

A cross-sectional study of 313,936 Arab adolescents aged 14–19 years between the years 2007–2022, and 289,616 adolescents in a matched Jewish comparison group.

**Results:**

The crude prevalence of comorbidities increased consistently from the ‘underweight’ to the ‘class 3 obesity’ category (from 0.24 to 6.41%, from 0.32 to 4.59%, and from 0.49 to 5.35% for HTN, DM2 and PCO, respectively). Compared to the reference ‘normal weight’ category, an incremental increase of aOR was observed by increasing weight category. The aORs for the ‘class 3 obesity’ category (95% CIs) were 26.00 (21.62–31.10), 10.82 (8.83–13.14), and 6.06 (95% CI 4.57–7.87) for HTN, DM2, and PCO, respectively. In the Jewish comparison group, lower aORs for HTN and DM2 were observed in the ‘class 3 obesity’ category. The increase in aORs with the increase in weight categories was more striking in cases of multiple comorbidities.

**Conclusion:**

The finding of a strong association of obesity severity with major cardiometabolic consequences in adolescence, as well as the unique ethnic features of these associations, can help focus national health initiatives on vulnerable adolescent groups.

## Introduction

Obesity is associated with cardiovascular risk factors, such as Hypertension (HTN), Diabetes Mellitus type 2 (DM2) and Polycystic ovaries (PCO) in adolescence (1–5). While adolescent obesity itself is associated with subsequent cardiovascular morbidity and mortality (6, 7), the presence of concomitant comorbidities further increases the risk (8–12).

These three conditions share common pathophysiological mechanisms, some of them, such as insulin resistance and compensatory hyperinsulinemia, hyperandrogenism, and activation of the renin-angiotensin system from hyperaldosteronism are well known (13, 14). Other mechanisms, such as increased levels of 20-hydroxyeicosatetraenoic acid, apelin, and polygenic predisposition have been proposed recently (15–17). Due to their common pathophysiology, these conditions frequently coexist (2, 18, 19). There are reports in the medical literature on racial and ethnic disparities in childhood HTN, DM2 and PCO for parameters including metabolic markers, phenotypic expression, and future consequences, as well as different associations with obesity and other comorbidities (20–24). A large cohort study from England reported that different BMI cutoffs were associated with the development of DM2 among different ethnicities, with lower cutoffs in minorities (25). Similarly, different associations of weight categories with metabolic consequences were demonstrated for different ethnicities in China, and even for various European nations (26, 27).

The topic of adolescent obesity and its association with concurrent and future morbidity has been studied extensively in Israel Jews. Most of these studies were based on height and weight measurements before mandatory military service, from which the Arab population is exempted (4–6, 28, 29).

As of December 2020, the Arab minority comprised more than 21% of the Israeli population with 1,956,000 citizens (30). Considering the possible ethnic differences in the cardiometabolic consequences of adolescent obesity and the lack of research in the Arab minority, we decided to focus our present study on this topic.

We examined associations between adolescent weight categories and HTN, DM2 and PCO, and combinations of them among Arab adolescents in Israel and compared them with a matched comparison group from the Israeli Jewish population to highlight any unique patterns of association for Arab adolescents.

## Methods

This was a retrospective cross-sectional study, based on the centralized computerized database of the Clalit Health Services (CHS), the largest health maintenance organization in Israel that provides healthcare services to approximately half of the Israeli population. The *study population* was comprised of all Arab adolescents aged 14–19 years who were CHS members and whose medical records contained measurements of height and weight during the study period between January 1, 2007 to December 31, 2022. We included a Jewish adolescent comparison group, with BMI measurements during the same period, matched by age, sex and socio-economic level to the study population.

### Data collection

The study database consisted of data obtained from the computerized centralized database of CHS. It included height and weight, BMI (and BMI percentile), socio-demographic data (age, sex, ethnic sector, district of residence, and three levels of socio-economic status based on participant’s residential zip code), the recorded diagnoses of interest, HTN, DM2, PCO, and major chromosomal anomalies and intellectual disabilities. Additionally, *the total number of insured Arab adolescents* aged 14–19 years during the study period was obtained to assess the rate of missing data. The Jewish comparison group was matched by gender and socio-economic status.

All methods were performed in accordance with relevant guidelines and regulations, such as data deidentification, and approval of the Organizational Review Board and the Ethics Committee (31). The Ethics Committee for Community-based Studies of Meir Medical Center, Kfar-Saba, Israel approved the study, and exempted it from having to sign informed consent forms.

### Definitions of variables

BMI was defined as weight in kilograms divided by height squared in meters. Adolescent weight categories were defined as percentiles determined by the U.S. Center for Disease Control and Prevention (CDC), which were validated for Israeli adolescents (32), as *‘underweight’* (BMI < 5th percentile)*, ‘normal weight’* (5th-84.9th percentile)*, ‘overweight’* (85th-94.9th percentile)*, ‘obesity’* (≥95th percentile, but not including ‘Class 2’ and ‘Class 3 obesity’) (33). *‘Class 2 obesity’* was diagnosed if BMI reached ≥120 to <140% of the 95th percentile or BMI ≥35 to <40 kg/m^2^. *‘Class 3 obesity’* was diagnosed if BMI ≥140% of the 95th percentile or BMI ≥40 kg/m^2^ (33). Importantly, validation of the CDC weight categories is available only for the Jewish, but not for the Arab population in Israel.

Since ICD-10 codes were introduced into the Israeli Healthcare System in 2013, and the study period extended between 2007 and 2022, we used the ICD-9 codes to ensure data uniformity as much as possible while extracting the relevant diagnoses (34). The diagnosis of *primary HTN* was established by an ICD-9 code of 401.0, 401.1, 401.9 and age of diagnosis ≥6 years, the youngest age in which primary hypertension usually develops (35). *DM2* was diagnosed by an ICD-9 code of 250.X0 or 250.X2 and the absence of an ICD-9 code of 250.X1 or 250.X3. This combination has shown a diagnostic accuracy of 100% and an area under the curve of 99.8% for the diagnosis of DM2 (36). Patients with *PCO* were defined by the ICD-9 code 256.4. *Major chromosomal and other congenital anomalies* were defined by ICD-9 codes of 758.0, 758.1, 758.2, 758.3, 758.5, 758.6, 758.7, 758.8, 758.9, 759.5, 759.6, 759.7, 759.8, 759.9, and *moderate to severe intellectual disabilities* by ICD codes 318, and 319.

It should be noted that the diagnostic criteria for all three diseases underwent changes during the study period. In 2017 a fixed cut-off of ≥130/80 mmHg independent of age, sex, and height was established for the diagnosis of HTN in adolescents, replacing the 95th percentile of blood pressure accepted before, which could have led to a slightly higher percent of diagnosed adolescents (37). As for DM2, in 2010 the ADA included HbA1C ≥ 6.5% as a criterion for diagnosis in adolescents, in addition to fasting plasma glucose above 126 mg/dL (≥ 7 mmol/L) and oral glucose tolerance test ≥200 mg/dL (11.1 mmol/L), with the aim of improving the specificity of the diagnosis (38). Regarding PCO, the change in 2018 to the less strict diagnostic criteria, which required ≥20 ovarian follicles on ultrasound instead of ≥12 follicles in the previous, Rotterdam criteria, could have decreased the number of females diagnosed with PCO (39). So slightly different populations of adolescents could have been diagnosed with these comorbidities during the study period by the criteria relevant at the time of diagnosis.

As the study period included 16 years, five consecutive 4-year birth cohorts from 1988–1991 to 2004–2008, were created to account for the possible effect of birth cohort on the obesity. The last birth cohort, 2004–2008, contained 5 years for technical reasons.

### Statistical analyses

Data cleaning was performed and outlying BMI values were deleted. We compared basic socio-demographic characteristics of the adolescents with and without BMI measurements to assess for possible selection bias. The study sample was completed after exclusion of patients with major chromosomal abnormalities and intellectual disabilities. As we aimed to examine the association of “having obesity at any point during adolescence” with comorbidities, we chose the maximal BMI measurement during the study period for each participant, for further analyses. We characterized the baseline features of the study population using descriptive statistics. We calculated the crude rates of comorbidities (HTN, DM2, PCO and any combination of them) in the different weight categories and compared them between the two ethnic groups. To assess the association of weight categories with the described comorbidities, we built logistic regression models with the categorical variable of weight category as the main independent variable, and the ‘normal weight’ category as the reference. We also examined the association of weight categories with combinations of comorbidities. Due to the low prevalence of these combinations in the sample we used the penalized logistic regression proposed by Firth (40) for samples with rare events. The models were adjusted for sex, district of residency, socio-economic status, and birth cohort. The models were applied separately to the Arab and Jewish groups and the effects were compared between them. Statistical analyses were conducted using the R software (version 4.3.2).

## Results

**Figure 1:**
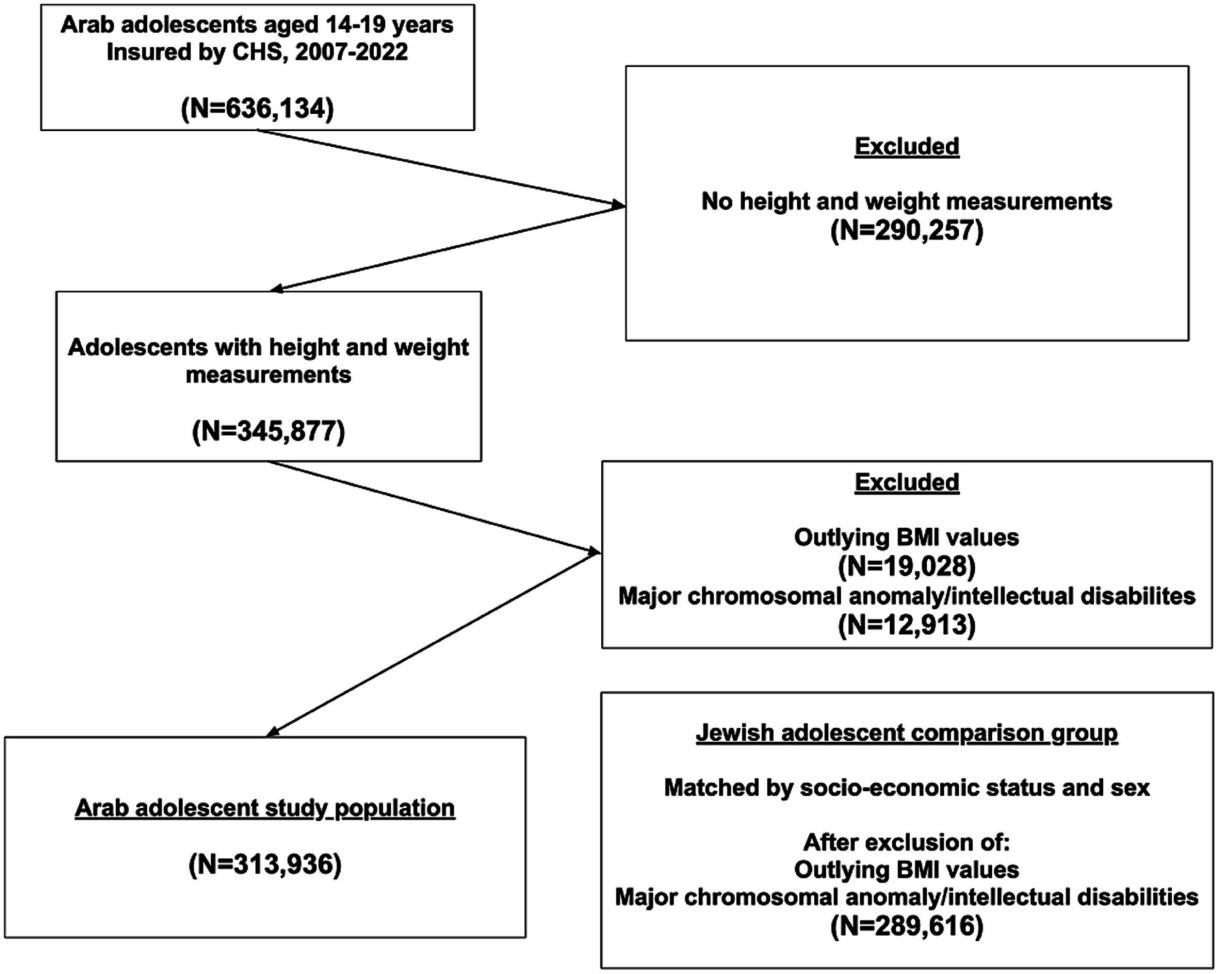
Flowchart of the selection process of study participants.

A flowchart of the selection process of the study participants is presented in Figure 1. Of 636,134 Arab adolescents insured in CHS during the study period, 345,877 had weight and height measurements in their medical records. After excluding 19,028 individuals with outlying BMI values and 12,913 cases of major chromosomal anomalies and moderate to severe intellectual disabilities, 313,936 adolescents aged 14–19 were included in the study. No major differences in socio-demographic characteristics (sex, socio-economic status, and district of residency) were seen between those with BMI measurements and those without BMI measurements, or with outlying BMI values. The Jewish comparison group, after exclusion of adolescents with intellectual disabilities and outlying BMI values, included 289,616 participants. Table 1 presents the socio-demographic characteristics of the adolescents. Males constituted 49.4% of the participants, only 0.6% were in the high class. The majority of Arab participants resided in Haifa district (23.5%), whereas the majority of Jews resided in Israel’s Central district (27.1%, *p* < 0.001). Many more Arab than Jewish participants belonged to the earlier 1988–1991 birth cohort, and many less to the recent 2004–2008 (15.0 vs. 10.8 and 6.2 vs. 14.6, respectively, *p* < 0.001). The percentage of adolescents in the ‘overweight’, ‘obesity’, ‘class 2’ and ‘class 3 obesity’ categories constituted 13.6, 13.6, 3.2, and 0.8% of the study participants among Arabs and 12.2, 13.9, 3.9, 1.3% among Jews, *p* < 0.001.

**Table 1:** Basic characteristics of study participants.

Variable	Arabs (*N* = 313,936)	Jews^a^ (*N* = 289,616)	*p*
District in Israel, N (%)			
Central	5,164 (1.64)	78,404 (27.07)	<0.001
Northern	62,988 (20.06)	37,893 (13.08)	
Haifa	73,623 (23.45)	44,384 (15.32)	
Sharon-Shomron	48,935 (15.59)	30,790 (10.63)	
Dan-PT	8,470 (2.70)	27,889 (9.63)	
Jerusalem	61,557 (19.61)	24,788 (8.56)	
Southern	53,199 (16.95)	45,425 (15.68)	
Missing	0 (0.0)	43 (0.01)	
Birth cohort, N (%)			
1988–1991	46,997 (14.97)	31,338 (10.82)	<0.001
1992–1995	83,558 (26.62)	75,981 (26.23)	
1996–1999	77,867 (24.80)	66,243 (22.87)	
2000–2003	86,106 (27.43)	73,850 (25.50)	
2004–2008	19,408 (6.18)	42,204 (14.57)	
Weight category, N (%)			
Underweight	9,944 (3.17)	14,089 (4.86)	<0.001
Normal	206,010 (65.62)	184,740 (63.79)	
Overweight	42,638 (13.58)	35,371 (12.21)	
Obesity	42,676 (13.59)	40,324 (13.92)	
Class 2 Obesity	10,141 (3.23)	11,416 (3.94)	
Class 3 Obesity	2,526 (0.80)	3,676 (1.27)	

^a^ Matched by sex (49.4% males) and socio-economic level (0.6% belonged to a high level). Underweight-BMI <5th percentile, normal weight-BMI 5th-84.9th percentile, overweight-BMI 85th-94.9th percentile, obesity-BMI ≥95th percentile, not including class 2 and class 3 obesity, class 2 obesity-BMI ≥120 to <140% of the 95^th^ percentile or BMI ≥35 to <40 kg/m^2^, class 3 obesity- BMI ≥140% of the 95^th^ percentile or BMI ≥40 kg/m^2^.

### Prevalence of HTN, DM2, PCO and combinations of them in different weight categories

**Figure 2:**
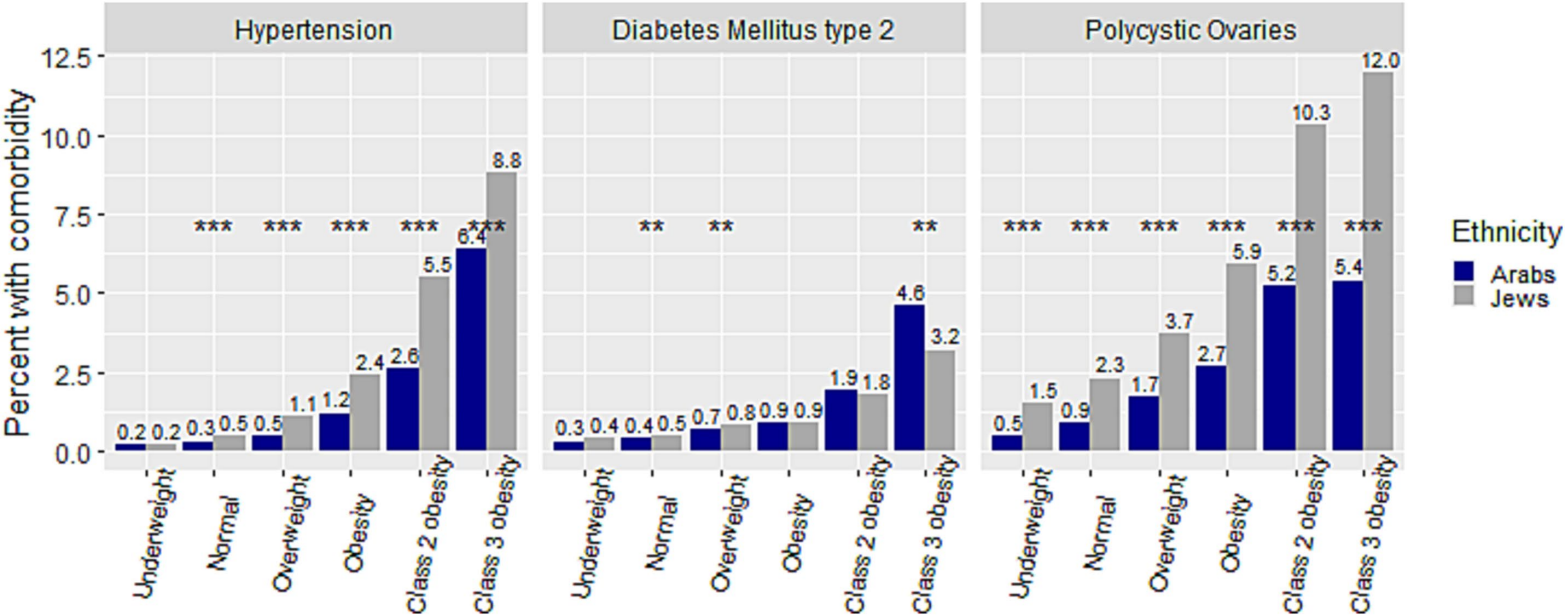
Comparison of percent of hypertension, diabetes mellitus type 2, and polycystic ovaries within weight categories between Arab and Jewish adolescents. **p < 0.01,***p < 0.001.

**Figure 3:**
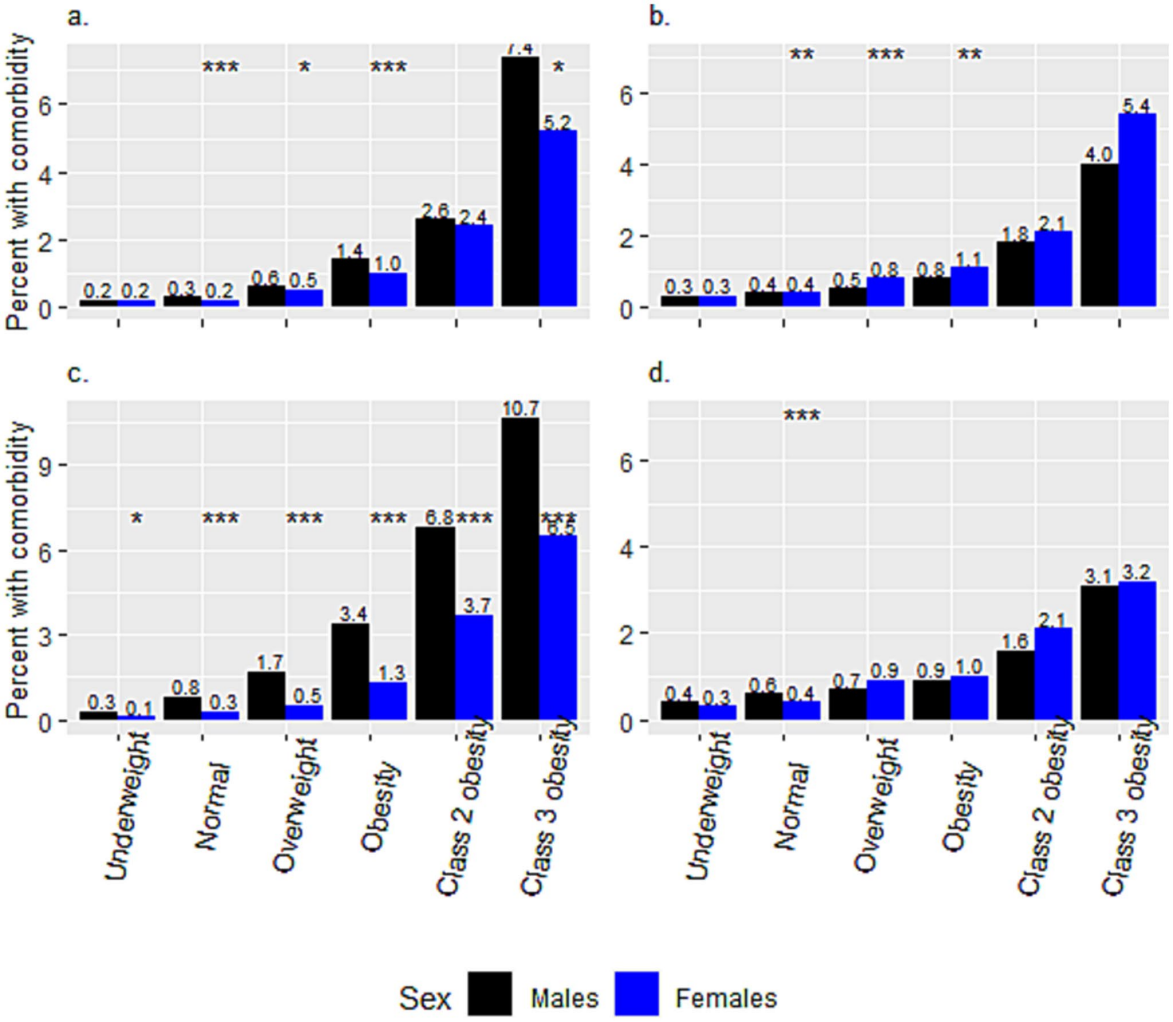
Sex differences in the percentages of hypertension and diabetes mellitus type 2, within weight categories, between Arab and Jewish adolescents. The prevalence of **(a)** Hypertension and **(b)** Diabetes Mellitus Type 2 among Arab adolescents. The prevalence of **(c)** Hypertension and **(d)** Diabetes Mellitus Type 2 among Jewish adolescents. *p < 0.05, **p < 0.01, ***p < 0.001.

Figure 2 presents a comparison of the crude prevalence of comorbidities among adolescents from different weight categories between the two ethnic groups. The crude prevalence of comorbidities for the Arab group increased incrementally from the ‘underweight’ to the ‘class 3 obesity’ category (from 0.24 to 6.41%, from 0.32 to 4.59%, and from 0.49 to 5.35%, for HTN, DM2 and PCO, respectively). The same increase for the Jewish group was from 0.21 to 8.84%, from 0.35 to 3.16%, and from 1.46 to 12.02%, for HTN, DM2 and PCO, respectively. Notably, the prevalence of PCO was significantly higher among Jewish than among Arab adolescent females in all weight categories, and that of HTN in all but the ‘underweight’ category, at *p* < 0.001. The prevalence of HTN was significantly higher among males than females in all but the ‘underweight’ and ‘class 2 obesity’ categories in Arabs, and in all weight categories in Jews (Figures 3A,C). The prevalence of DM2 was significantly higher among females than males in the ‘normal’, ‘overweight’, and ‘obesity’ categories among Arab adolescents, but only in the ‘normal’ weight category among Jewish (Figures 3B,D).

Additional data on the prevalence of comorbidities and their combinations within different weight categories are presented in Supplementary material 1. The prevalence of combinations of comorbidities increased with the increase in weight category as well, with a steep increase in the ‘class 2’ and ‘class 3’ obesity categories. The increase from ‘underweight’ to ‘class 3 obesity’ categories among the Arab adolescents was from 0.03 to 1.15%, 0.00 to 0.82%, and 0.00 to 0.45% for the combinations of HTN and DM2, DM2 and PCO, and HTN and PCO, respectively. Among the Jewish adolescents the prevalence of these combinations was similar across weight categories, with the single exception of a significantly higher rate of PCO and HTN and PCO and DM2 in the ‘normal’ weight category (*p* < 0.001; Supplementary material 1).

### Associations of weight categories with HTN, DM2 and PCO and comparison with Jewish counterparts

**Figure 4:**
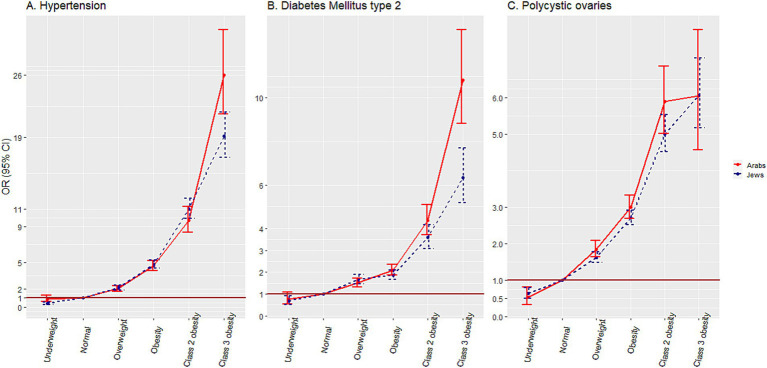
Associations of weight categories with hypertension, diabetes mellitus and polycystic ovaries, among adolescents.

Figure 4 and Supplementary material 2 present the association of weight categories with comorbidities, adjusted for sex, socio-economic status, birth cohort, and district of residency. The association with PCO was calculated among females, and adjusted for socio-economic status, birth cohort and district of residency only. As compared to the reference ‘normal weight’ category, an incremental increase of aOR for HTN was observed with increase in weight category from aOR = 2.06 (95% CI 1.76–2.40) for ‘overweight’ to aOR = 26.00 (95% CI 21.62–31.10) for the ‘class 3 obesity’ category. The same trend was observed for DM2, where, as compared to the reference ‘normal’ weight category, the aOR increased from aOR = 1.51 (95% CI 1.32–1.73) to aOR = 10.82 (95% CI 8.83–13.14) in the ‘overweight’ and the ‘class 3 obesity’, respectively. Regarding PCO, in addition to the increase in aOR from aOR = 1.85 (95% CI 1.64–2.09) to aOR = 6.06 (95% CI 4.57–7.87) in the ‘overweight’ and the ‘class 3 obesity’, respectively, the aOR was lower compared to the reference group in the ‘underweight’ category aOR = 0.54 (95% CI 0.33–0.81).

In the Jewish comparison group, lower aORs for HTN and DM2 were observed in the ‘class 3 obesity’ category [aOR = 19.17 (95% CI 16.77–21.86)] and aOR = 6.35 (95% CI 5.19–7.69), respectively. The degree of association of the weight categories with PCO was similar in the two ethnic groups.

Supplementary materials 2, 3 present the associations of weight categories with combinations of comorbidities. The increase in aORs with the increase in weight categories was even more impressive, reaching aORs (95% CIs) of 113.37 (64.47–194.50), 85.97 (26.06–273.55), and 126.49 (48.14–344.59) in ‘class 3 obesity’ for the combinations of HTN and DM2, PCO and HTN and PCO and DM2, respectively. These associations were similarly strong in the Jewish comparison group.

## Discussion

In this study of 313,936 Arab adolescents, we demonstrated a strong association between weight categories and comorbidities (HTN, DM2, and PCO).

The overall prevalence of HTN and the increase in its prevalence among weight categories was similar to the results of previous studies (41–43). In other studies (4, 44, 45), which reported the same increase pattern across weight categories, the crude rates of HTN were much higher than in the present study, probably due to the different case definitions, using active surveillance of the disease in contrast to recorded physician diagnoses as in our study. The increase in prevalence was higher in males than in females, as in most other studies (4, 42, 44, 45). A higher prevalence rate of HTN was reported previously for Jewish compared to the Arab adult population (46). Our study revealed that this discrepancy begins already in adolescence. The aORs for HTN among excess weight categories, which reached an aOR = 26.00 (95% CI 21.62–31.10) for the ‘class 3 obesity’ category, were higher than other reports from around the world (41–44, 47), higher than reported for Jewish adolescents from Israel (4), and higher than in the Jewish comparison group in our study. Reports on different associations of obesity severity with HTN among adolescents from different ethnic groups appeared recently in several studies from the United States (41–43).

An increase in the prevalence of DM2 across weight categories was higher than reported in the past in a Jewish adolescent population in Israel (4), but lower than reported from the United States (42). In contrast to HTN, a clear females preponderance was observed for DM2 in the ‘normal’, ‘overweight’, and ‘obesity’ weight categories among Arabs, and in the ‘normal’ weight category among Jews. This finding is in line with the report for Jewish youth in Israel (4), and is opposite to the findings from the United States, where boys had an OR of 2.5 for high blood sugar (43). The high prevalence of DM2 among Arabs in the ‘class 3 obesity’ category, which exceeded that among Jewish adolescents, was reflected in the steep increase in aORs for DM2 for ‘class 3 obesity’, which was higher than that reported in other studies (42, 43), and in the Jewish comparison group in our study. Recent reports from USA demonstrated higher odds for DM2 for Hispanic youth compared to non-Hispanic White (42, 43).

Although other studies on the adolescent obesity and PCO (48, 49) did not distinguish between ‘class 2’ and ‘class 3 obesity’, the reported prevalence of the condition among Arab adolescents with obesity was very similar to that found in our study. Jews had consistently higher prevalence rates of PCO than Arabs, which is not surprising, given the well-known high prevalence of this syndrome among Jews (50). The increase in aORs to 5.89 (95% CI 5.02–6.87) in the ‘class 3 obesity’ category, was nearly identical to the increase demonstrated in a study from Denmark (48) and lower than in a study from United States (49). No major ethnic differences in the association of PCO with obesity severity were found in our study, in contrast to a study that found significant differences in this association among ethnic groups in the United States (49).

The magnitude of the association of obesity severity with the combinations of comorbidities is of special note, and to our knowledge was not assessed in previous studies.

### Limitations and strengths

The first limitation of our study is missing data on BMI in medical records, which may have led to selection bias. On the other hand, the recommendation to measure height and weight for BMI, which applies to all adolescents aged 14–19 years, has been an integral part of the Israel National program for quality indicators in the community since 2007. Thus, missing BMI measurements could be considered as randomly missing data. Furthermore, we demonstrated similarity in the socio-demographic characteristics between the adolescents with and without measurements in our study. The second limitation is that the data on the investigated comorbidities were based on recorded diagnoses, which might have underestimated the effect measures. Medical surveillance could have yielded more accurate case definitions, although this approach was not possible in a study based on a large nationwide database. Furthermore, due to the changes in diagnostic criteria, slightly different populations of adolescents could have been diagnosed with comorbidities at different points in time during the study period. In addition, the cross-sectional study design did not allow us to assess causality, only associations. The specific ethnic nature of the study population limits its generalizability, and it may be primarily of local importance. The main strength of this study is that it was based on a nationwide, representative, large and reliable database, with a high number of observations. Another strength is that the weight and height measurements and diagnoses were recorded by health care professionals and not self-reported. The allocation to ‘class 2’ and ‘class 3’ obesity categories made it possible to identify associations specific to these extreme degrees of obesity.

## Conclusion

Given the recent reports on an increased prevalence of adolescent obesity in Israel (51) and its high prevalence in the Arab minority (52), it is crucial to recognize and elucidate associations of obesity with chronic morbidity. The evidence that obesity severity is associated with major cardiometabolic consequences in adolescence, as well as the unique ethnic features of these associations, such as the stronger associations of ‘class 2’ and ‘class 3 obesity’ with HTN and DM2 in Arab adolescents compared to their Jewish counterparts, further highlights the need for national health initiatives to tackle the problem of adolescent obesity and help focus on interventions designed for these vulnerable adolescent groups.

## Data Availability

The datasets presented in this article are not readily available because the data that support the findings of this study are available from ‘Clalit Health Services’. Restrictions apply to the availability of these data, which were used under license for this study. The data are available only with the permission of the ‘Clalit Health Services’. Requests to access the datasets should be directed to Yulia Treister-Goltzman, yuliatr@walla.com.
