# Supplementary material for: Associations of adolescent obesity with hypertension, diabetes mellitus and polycystic ovaries in Arabs and Jews in Israel—a nationwide study
Source: Front Public Health. 2024 Dec 12;12:1443756. doi: 10.3389/fpubh.2024.1443756 (PMC11669581; doi:10.3389/fpubh.2024.1443756)
Supplement: Supplementary file 1 [file Table_1.docx]

**Online Resource 1.** Cases of Diabetes Mellitus type 2, Hypertension, Polycystic ovaries and their combinations within different weight categories

|  | CDC BMI category | | | | | | | | | | | |
| --- | --- | --- | --- | --- | --- | --- | --- | --- | --- | --- | --- | --- |
| Comorbidity | Underweight | | Normal | | Overweight | | Obesity | | Class 2 Obesity | | Class 3 Obesity | |
|  | Arabs (N=9,944) | Jews  (N=14,089) | Arabs (N=206,010) | Jews (N=184,740) | Arabs (N=42,638) | Jews (N=35,371) | Arabs (N=42,676) | Jews (N=40,324) | Arabs (N=10,142) | Jews (N=11,416) | Arabs (N=2,526) | Jews (N=3,676) |
| *HTN*  N (%)  Both sexes  Males  Females | 24 (0.24)  14 (0.24)  10 (0.24) | 30 (0.21)  25 (0.29)  5 (0.09) | 537 (0.26)^***^  306 (0.31)^***^  231 (0.21)^***^ | 952 (0.51)  676 (0.75)  276 (0.29) | 230 (0.54)^***^  132 (0.63)^***^  98 (0.46) | 387 (1.09)  297 (1.69)  90 (0.51) | 515 (1.21)^***^  323 (1.40)^***^  192 (0.98)^**^ | 971 (2.41)  727 (3.44)  244 (1.27) | 260 (2.56)^***^  166 (2.64)^***^  94 (2.44)^**^ | 624 (5.47)  445 (6.77)  179 (3.70) | 162 (6.41)^***^  105 (7.38)^**^  57 (5.17) | 325 (8.84)  219 (10.70)  106 (6.50) |
| *DM2*  N (%)  Both sexes  Males  Females | 32 (0.32)  18 (0.31)  14 (0.34) | 50 (0.35)  32 (0.37)  18 (0.32) | 885 (0.43)^**^  454 (0.47)^**^  431 (0.40)^***^ | 926 (0.50)  518 (0.57)  408 (0.43) | 277 (0.65)^**^  110 (0.52)^**^  167 (0.78) | 293 (0.83)  131 (0.74)  162 (0.91) | 389 (0.91)  178 (0.77)  211 (1.08) | 378 (0.94)  179 (0.85)  199 (1.04) | 191 (1.88)  110 (1.75)  81 (2.10) | 207 (1.81)  106 (1.61)  101 (2.09) | 116 (4.59)^**^  57 (4.01)  59 (5.35)^**^ | 116 (3.16)  63 (3.08)  53 (3.2) |
| *PCO*  N (%)  Females | 20 (0.49) | 81 (1.46) | 993 (0.91)^***^ | 2,202 (2.33) | 363 (1.69)^***^ | 657 (3.70) | 531 (2.71)^***^ | 1,135 (5.92) | 199 (5.16)^***^ | 496 (10.25) | 59 (5.35)^***^ | 196 (12.02) |
| *Combination of DM2 and HTN*  N (%)  Both sexes  Males  Females | 3 (0.03)  3 (0.05)  0 (0.00) | 0 (0.00)  0 (0.00)  0 (0.00) | 23 (0.01)  11 (0.01)  12 (0.01)^***^ | 18 (0.01)  10 (0.02)  8 (0.01) | 11 (0.03)  4 (0.02)  7 (0.03) | 8 (0.02)  5 (0.03)  3 (0.02) | 27 (0.06)  8 (0.03)  19 (0.10) | 24 (0.06)  16 (0.08)  8 (0.04) | 28 (0.28)  13 (0.21)  15 (0.39) | 38 (0.33)  19 (0.29)  19 (0.39) | 29 (1.15)^*^  13 (0.91)  16 (1.45) | 23 (0.63)  12 (0.59)  11 (0.67) |
| *Combination of DM2 and PCO*  N (%)  Females | 0 (0.00) | 0 (0.00) | 7 (0.01)^***^ | 10 (0.01) | 9 (0.04) | 6 (0.03) | 14 (0.07)^**^ | 33 (0.17) | 7 (0.18)^*^ | 24 (0.50) | 9 (0.82) | 17 (1.04) |
| *Combination of HTN and PCO*  N (%)  Females | 0 (0.00) | 0 (0.00) | 6 (0.01)^***^ | 8 (0.01) | 3 (0.01) | 3 (0.02) | 12 (0.06) | 8 (0.04) | 9 (0.23) | 19 (0.39) | 5 (0.45) | 11 (0.67) |

Underweight- BMI <5th percentile, normal weight- BMI 5th-84.9th percentile, overweight- BMI 85th-94.9th percentile, obesity- BMI ≥95th percentile, not including class 2 and class 3 obesity, class 2 obesity- BMI ≥120% to <140% of the 95^th^ percentile or BMI ≥35 to <40 kg/m^2^, class 3 obesity- BMI ≥140% of the 95^th^ percentile or BMI ≥40 kg/m^2^. HTN- Hypertension, DM2- Diabetes Mellitus type 2, PCO- Polycystic ovaries. ^***^Differs from the Jewish group at p<0.001, ^**^differs from the Jewish group at p<0.01, ^*^differs from the Jewish group at p<0.05
