# Supplementary material for: Associations of adolescent obesity with hypertension, diabetes mellitus and polycystic ovaries in Arabs and Jews in Israel—a nationwide study
Source: Front Public Health. 2024 Dec 12;12:1443756. doi: 10.3389/fpubh.2024.1443756 (PMC11669581; doi:10.3389/fpubh.2024.1443756)
Supplement: Supplementary file 2 [file Table_2.docx]

**Online Resource 2.** Associations of weight categories with Hypertension, Diabetes Mellitus type 2, Polycystic ovaries, and their combinations among Arab adolescents and Jewish comparison group.

| Comorbidity | CDC Weight Categories | | | | | | | | | | |
| --- | --- | --- | --- | --- | --- | --- | --- | --- | --- | --- | --- |
|  | Underweight | | Normal weight | Overweight | | Obesity | | Class 2 obesity | | Class 3 obesity | |
|  | ***Arab adolescents*** | | | | | | | | | | |
|  | OR | CI (95%) | Reference | OR | CI (95%) | OR | CI (95%) | OR | CI (95%) | OR | CI (95%) |
| Hypertension | 0.91 | 0.59- 1.33 |  | 2.06*** | 1.76-2.40 | 4.60*** | 4.07-5.19 | 9.72*** | 8.35-11.29 | 26.00*** | 21.62-31.10 |
| Diabetes Mellitus | 0.78 | 0.54- 1.10 |  | 1.51*** | 1.32-1.73 | 2.10*** | 1.86-2.37 | 4.37*** | 3.72-  5.11 | 10.82*** | 8.83-13.14 |
| Polycystic ovaries | 0.54** | 0.33-0.81 |  | 1.85*** | 1.64-2.09 | 3.00*** | 2.69-3.33 | 5.89*** | 5.02-  6.87 | 6.06*** | 4.57-  7.87 |
| Combination of Hypertension and Diabetes Mellitus type 2 | 3.49 | 0.93-9.49 |  | 2.40* | 1.14-4.77 | 6.00*** | 3.45-10.50 | 27.40*** | 15.81-47.95 | 111.37*** | 64.47-194.50 |
| Combination of Polycystic ovaries and Hypertension | 2.08 | 0.02- 17.58 |  | 2.75 | 0.65-9.67 | 10.87*** | 4.33-30.05 | 41.71*** | 15.48-119.70 | 85.97*** | 26.06-273.55 |
| Combination of Polycystic ovaries and Diabetes Mellitus type 2 | 1.80 | 0.01-14.81 |  | 6.37*** | 2.44-17.22 | 10.86*** | 4.61-27.79 | 29.35*** | 10.41-82.77 | 126.49*** | 48.14-344.59 |
|  | ***Jewish comparison group*** | | | | | | | | | | |
| Hypertension | 0.38*** | 0.26-0.54 |  | 2.15*** | 1.91-2.42 | 4.78*** | 4.36-5.23 | 11.00*** | 9.91-12.20 | 19.17*** | 16.77-21.86 |
| Diabetes Mellitus | 0.69*** | 0.51-0.91 |  | 1.66*** | 1.45-1.89 | 1.87*** | 1.66-2.11 | 3.61*** | 3.09- 4.19 | 6.35*** | 5.19- 7.69 |
| Polycystic ovaries | 0.64*** | 0.50-0.79 |  | 1.62*** | 1.48-1.77 | 2.71*** | 2.52-2.92 | 5.00*** | 4.51-5.54 | 6.07*** | 5.18-7.08 |
| Combination of Hypertension and Diabetes Mellitus type 2 | 0.35 | 0.00-2.59 |  | 2.45* | 1.03-5.37 | 6.38*** | 3.50-11.82 | 35.98*** | 20.90-64.19 | 69.29*** | 37.61-129.52 |
| Combination of Polycystic ovaries  and Hypertension | 0.83 | 0.01-6.47 |  | 3.36* | 1.19-8.76 | 16.69*** | 8.64-35.06 | 49.98*** | 24.86-107.69 | 111.60*** | 52.39-249.40 |
| Combination of Polycystic ovaries and Diabetes Mellitus type 2 | 1.04 | 0.01-8.34 |  | 2.25 | 0.56-7.29 | 5.29*** | 2.01-13.93 | 49.19*** | 22.58-116.61 | 89.73*** | 36.86-227.03 |

Underweight- BMI <5th percentile, normal weight- BMI 5th-84.9th percentile, overweight- BMI 85th-94.9th percentile, obesity- BMI ≥95th percentile, not including class 2 and class 3 obesity, class 2 obesity- BMI ≥120% to <140% of the 95^th^ percentile or BMI ≥35 to <40 kg/m^2^, class 3 obesity- BMI ≥140% of the 95^th^ percentile or BMI ≥40 kg/m^2^, OR-Odds Ratio, CI (95%)-confidence interval (95%) of the Odds Ratio; ***p<0.001, *p<0.05
