# Supplementary material for: Associations of adolescent obesity with hypertension, diabetes mellitus and polycystic ovaries in Arabs and Jews in Israel—a nationwide study
Source: Front Public Health. 2024 Dec 12;12:1443756. doi: 10.3389/fpubh.2024.1443756 (PMC11669581; doi:10.3389/fpubh.2024.1443756)
Supplement: Supplementary file 3 [file Table_3.docx]

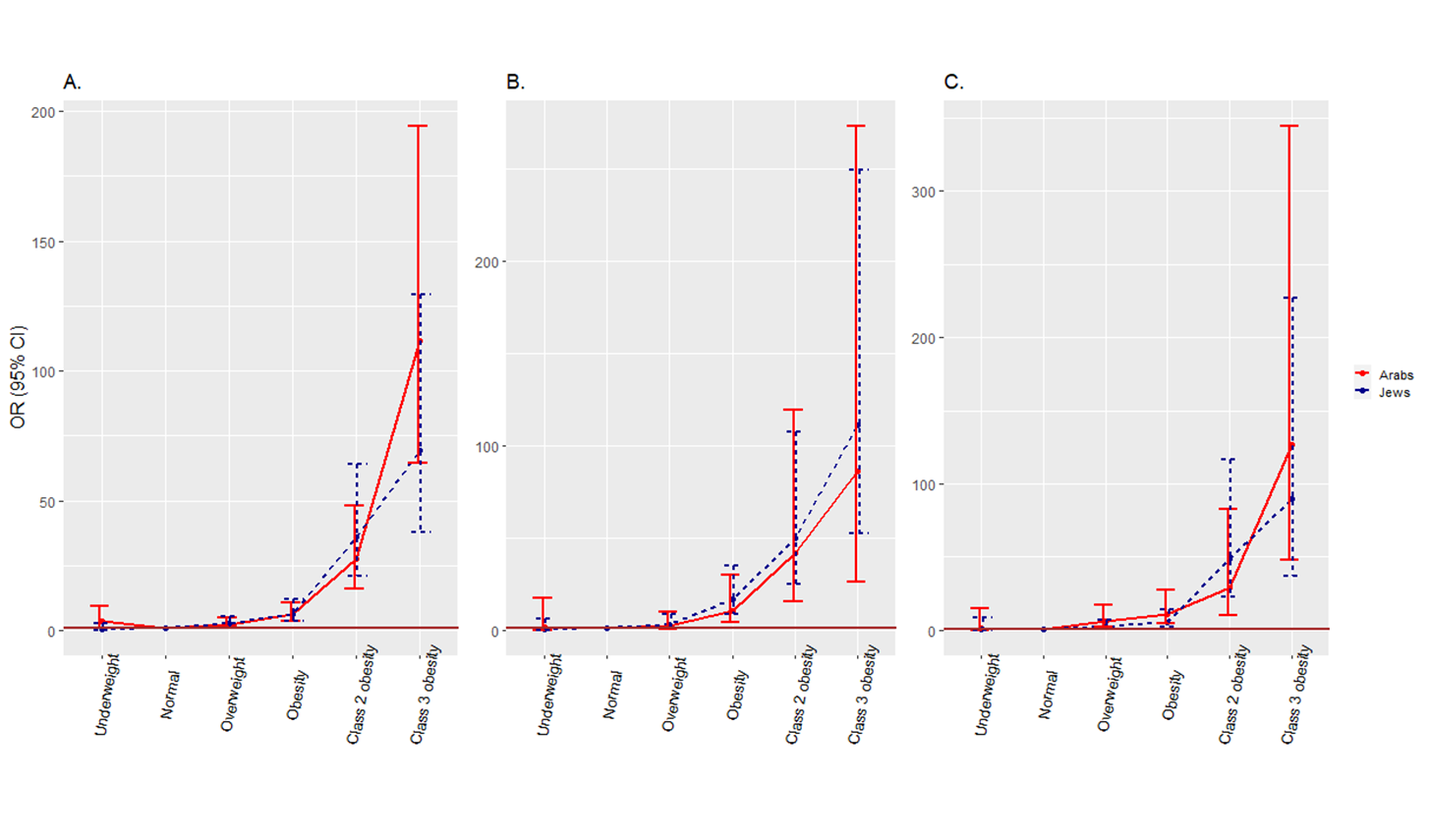
**Online Resource 3.** Associations of weight categories with combinations of comorbidities among Arab and Jewish adolescents

A- combination of Hypertension and Diabetes Mellitus type 2, B- combination of Polycystic ovaries with Hypertension among females, C- combination of Polycystic ovaries with Diabetes Mellitus type 2 among females. Underweight- BMI <5th percentile, normal weight- BMI 5th-84.9th percentile, overweight- BMI 85th-94.9th percentile, obesity- BMI ≥95th percentile, not including class 2 and class 3 obesity, class 2 obesity- BMI ≥120% to <140% of the 95^th^ percentile or BMI ≥35 to <40 kg/m^2^, class 3 obesity- BMI ≥140% of the 95^th^ percentile or BMI ≥40 kg/m^2^.
